# The Upsides and Downsides of High Self-Control: Evidence for Effects of Similarity and Situation Dependency

**DOI:** 10.5964/ejop.2639

**Published:** 2021-02-26

**Authors:** Lukas Röseler, Jacqueline Ebert, Astrid Schütz, Roy F. Baumeister

**Affiliations:** a Department of Personality Psychology and Psychological Assessment, University of Bamberg, Bamberg, Germany; b Department of Economics, Harz University of Applied Sciences, Wernigerode, Germany; c School of Psychology, University of Queensland, Brisbane, Australia; University of Palermo, Palermo, Italy

**Keywords:** self-control, social perception, attraction

## Abstract

High trait self-control is generally depicted as favorable. We investigated whether this holds for social perception. Using vignettes, we tested whether a person with high self-control is 1) preferred as a partner for all or only certain social situations, 2) perceived as less likeable than a person with low self-control, 3) liked more if the person is female and the behavior thus fits the sex-stereotype, and 4) perceived differently from a person with low self-control with respect to a wide range of adjectives used to describe personality. Competing theories are presented for each area. Results indicate that although high self-control is associated with a wide range of socially desirable traits, choice of partners 1) depends on the type of situation in which the interaction will occur, 2) depends on the similarity between the respondent and the partner, 3) does not depend on a stereotype match, and 4) does not depend or depends only to a small degree on the partner's high self-control. The perception of individuals with high self-control is thus variable and situationally contingent, and more than a single theory is needed to explain it.

Self-control, which is defined as people's capacity to alter their own responses and bring them in line with things such as social expectations, is generally considered a socially desirable characteristic (e.g., Baumeister, Vohs, & Tice, 2007). Indeed, even the measurement of self-control invokes the assumption of benefits. One self-control item is “I do certain things that are bad for me...” (reverse scored; Tangney, Baumeister, & Boone, 2004). In fact, it has been shown that self-control is advantageous in many areas of life, such as subjective well-being, binge-eating, alcohol use, grades in school, commitment in a relationship (de Ridder, Lensvelt-Mulders, Finkenauer, Stok, & Baumeister, 2012), malevolent and fractious intentions, outward directed aggression (Tangney et al., 2004), or selfishness and helpfulness (Dewall, Baumeister, Gailliot, & Maner, 2008). In a way, self-control implies restraining bodily urges that include short-term pleasure. Thus, there is an assumption that people will act in selfish and reward- or lust-oriented ways (e.g., the selfishness hypothesis of ego depletion; Banker, Ainsworth, Baumeister, Ariely, & Vohs, 2017) when no superordinate entity is present to exert control over them (e.g., Hobbes & Brooke, 1651/2017). Self-control would thus be fundamentally and pervasively beneficial. We refer to this as the positivity hypothesis, that is, the idea that high self-control is either beneficial or irrelevant. Like intelligence, self-control can be compared to a tool. Tools are good to have. To be sure, tools can be used for bad ends, but that does not reflect any fault of the tools themselves.

Past research has occasionally looked for disadvantages of high self-control but without much success. For example, no support was found for the hypothesis that too much self-control (i.e., a curvilinear relationship between self-control and other variables, so that intermediate levels of self-control produce the best results) could contribute to compulsions and eating disorders. Instead, Tangney et al. (2004) found a linear negative relationship between self-control and the occurrence of impulse control problems (e.g., binge eating) and self-reports of psychopathological symptoms (e.g., obsessive-compulsive patterns, anxiety). About the worst that can be said about high self-control is that individuals with high self-control tend to be confronted with higher expectations and are therefore more likely to suffer from exhaustion (Koval, vanDellen, Fitzsimons, & Ranby, 2015) and that high functional impulsivity (a variation of low self-control) can be advantageous when there is a need to process information quickly (Dickman & Meyer, 1988).

Even less attention has been devoted to whether interpersonal problems are related to high self-control, even though striving and conformity are not necessarily reflected in positive relationships. For example, students who get good grades in school are more likely to be labeled nerds, which is a slang term that disparages scholarly achievement (Pelkner & Boehnke, 2003; Rentzsch, Schütz, & Schröder-Abé, 2011). Even so, such stigmatizing seems to reflect anti-intellectual resentment more than a genuine social disadvantage of high self-control. Much work has confirmed that people with high self-control are more popular and have better relationships than other people (e.g., Maszk, Eisenberg, & Guthrie, 1999; Vohs, Finkenauer, & Baumeister, 2011).

## Derivation of Hypotheses

In the present study, we took a social perception perspective to investigate whether there may be interpersonal downsides of high trait self-control. To cover a broad area, we included 1) preferences for partners with high versus low self-control in various social situations, 2) liking and stereotypes of people with high versus low self-control, and 3) perceivers' personality traits.

## Downsides of High Self-Control in Social Interactions

Assuming that a person with low self-control agrees with the statement “I do certain things that are bad for me, if they are fun” (Tangney et al., 2004), that person might be considered good company if there is a lively party with lots of food and drink. Such activities are common: Among Germans between 18 and 25 years of age, 33.4% report engaging in the regular consumption of alcohol (BZgA, 2019). Thus, being self-controlled may interfere with being perceived as good company during leisure time and at parties.

Of course, everyday situations differ with regard to the kinds of behavior that are expected. Taxonomies such as the one by Rauthmann et al. (2014) have distinguished types of situations such as sociality and duty. Duty situations typically feature aspects that are not fun but need to be done (e.g., doing homework or cleaning the house). Socializing situations are often hedonic (e.g., going to a party or cooking a meal together). We hypothesized that whether people would prefer the company of a person with high or low self-control would depend on the situation they are in. Note that Lösch and Rentzsch (2018) found that students like to *work* with partners with high conscientiousness (which overlaps with self-control), but during leisure time, people with high conscientiousness are not chosen over people with low conscientiousness.

We propose three competing hypotheses with regard to the interaction of self-control and situations. Our thinking invoked Mischel's (1968) distinction between strong and weak situations (see also Cooper & Withey, 2009). In this understanding, strong situations leave little or no room for different behaviors (e.g., everyone stops at a red light when driving a car) whereas weak situations leave much room (e.g., people behave differently during a private party). The hypotheses thus differ with respect to how strongly the situation (duty vs. socializing) impacts preferences for an interaction partner with high versus low self-control.

1. Strong situation hypothesis: In situations that call for duty, people prefer to be with a person with high self-control rather than a person with low self-control. In socializing situations, people prefer to be with a person with low self-control rather than a person with high self-control (high situational strength for duty and socializing).
2. Weak situation hypothesis: In duty situations, people prefer to be with a person with high self-control rather than a person with low self-control. In socializing situations, people have no preference (high situational strength for duty and low situational strength for socializing).
3. Positivity hypothesis: In any type of situation, people prefer to be with a person with high self-control rather than a person with low self-control (no situational strength).

## Perceivers' Traits in Relation to Targets' Traits

One of the main predictors of whether a person likes another person is similarity (Byrne & Nelson, 1965). The so-called similarity hypothesis has received a lot of support with regard to personality traits such as optimism in romantic relationships (Böhm, Schütz, Rentzsch, Körner, & Funke, 2010), personality and physical attractiveness in roommate relationships (Carli, Ganley, & Pierce-Otay, 1991), humor style in married couples (Hahn & Campbell, 2016), gender-role self-concept and selection of dating partners (Morell, Twillman, & Sullaway, 1989), socially undesirable traits such as Machiavellianism (Ináncsi, Láng, & Bereczkei, 2016), and same-sex friendships regardless of duration or closeness (Morry, 2005). The meta-analytic effect of interpersonal attraction and perceived similarity was estimated to be *r* = .39 (Montoya, Horton, & Kirchner, 2008). However, hypotheses that propose effects that go in the opposite direction have also been formulated: According to the complementarity hypothesis, which is based on the circumplex theory (Kiesler, 1983), relationships work best when the two partners have different scores with regard to dominance but not with regard to affiliation. This finding was supported with respect to dependent variables such as liking in the context of dominant and submissive nonverbal behaviors (Dryer & Horowitz, 1997; Tiedens & Fragale, 2003) or relationship quality and facial attractiveness (Asendorpf, Penke, & Back, 2011, p. 25, Hypothesis 3b), but it was also challenged by null findings (Asendorpf et al., 2011, p. 25). Boman (2016) found that friends' levels of self-control are largely unrelated in both attitudinal and behavioral measures. With regard to self-control, Vohs et al. (2011) found that romantic couples seemed to pair up in a complementary fashion, such as that dating partners were less similar to each other than they would be by chance (p. 143). The effect was weaker (but still significant) among married couples and absent among same-sex friends, suggesting that the attraction was primarily sexual. Neither similarity nor complementarity predicted relationship satisfaction, which was simply (and positively) correlated with the total of both partners' self-control, consistent with the positivity hypothesis.

Third, there is the compensation hypothesis, according to which qualities of one partner contribute to the other's satisfaction and may even partially compensate for the partner's lack of these traits (Schröder-Abé & Schütz, 2011). Thus, individuals with low self-control may prefer individuals with high self-control to compensate for their own lack of self-control. Shea, Davisson, and Fitzsimons (2013) provided evidence for such effects with respect to self-control and confirmed the compensation hypothesis when they tested it against the other theories for both trait and state self-control. However, compensation worked for low self-controlled individuals only. That is, low self-controlled individuals preferred high self-controlled partners but high self-controlled partners had no preference.

On the basis of these models, we hypothesized that liking would be a function of people's own trait self-control and the trait self-control described in the vignettes, a tendency that has been observed for optimism (Böhm et al., 2010). Specifically, we derived the following competing hypotheses:

1. Similarity hypothesis: People with high self-control will express greater liking for other people with high self-control than for people with low self-control. People with low self-control will express greater liking for other people with low self-control than for people with high self-control.
2. Complementarity hypothesis: People with high self-control will express lower liking for other people with high self-control than for people with low self-control. People with low self-control will express lower liking for other people with low self-control than for people with high self-control.
3. Compensation hypothesis: People with low self-control will express greater liking for people with high self-control than for other people with low self-control. However, people with high self-control will express equally high levels of liking for people with high or low self-control.
4. Positivity hypothesis of self-control: Everyone will express greater liking for people with high self-control than for people with low self-control.

Moreover, liking has been found to depend on how well a person fits a stereotype: Probably the most salient category regarding stereotypes is the target person's sex (e.g., Hertel, Schütz, DePaulo, Morris, & Stucke, 2007). For example, anger is perceived more strongly in men than in women, whereas the opposite pattern has been found for sadness (Plant, Kling, & Smith, 2004). As men are stereotypically viewed as more aggressive than women and aggression is linked to losing one's temper, high aggressiveness may correspond with low self-control (Tangney et al., 2004). In line with this argument, men were found to display more explosive than defusing behavior (Campbell & Muncer, 2008). According to the German Federal Statistics Office, the perpetrators of accidents that involve injuries to people caused by people who are under the influence of alcohol or drugs are comprised of 87% men and 13% women. The same has been found for speeding (78% men; Statistisches Bundesamt, 2019). Thus, compared with women, men should be perceived as having less self-control. We hypothesized that conforming to this stereotype would boost liking. Accordingly, we propose the following sex-stereotype hypothesis.

Sex-stereotype hypothesis: Women with high self-control will be liked more than men with high self-control.

## Halo Effects of High Self-Control

How much people like a person or how much they would like to be with him or her in a specific situation might not only depend on the person's apparent self-control. Halo effects (e.g., Nisbett & Wilson, 1977) suggest that high trait self-control, a trait that is generally evaluated in a positive manner, could lead people to assume that the person possesses other desirable traits, which in turn may influence how much they like the person. We used the continuum model (Fiske & Neuberg, 1990) to explore which traits may be seen as related to a person's trait self-control. The model is broad as it consists of eight scales comprised of adjectives that describe people and that can be arranged in terms of how much control a person exerts over others (i.e., assured-dominant vs. unassured-submissive) and how strongly a person strives for affiliation (i.e., cold-hearted vs. warm-agreeable).

As yet, only a few social perception studies investigated burdens of high self-control. For example, high self-control people are burdened by others' reliance (Koval et al., 2015). Fairness in supervisors (Cox, 2000; as cited by Tangney et al., 2004) and trust (Righetti & Finkenauer, 2011) are affected positively by high self-control. There was no evidence of negative effects of self-control in either of the studies; instead, high self-control was *positively* associated with perceived fairness (as found in the study by Cox) and trust (as found in both studies). There have been similar findings involving school relationships: High-achieving students who probably have high self-control were also regarded as being highly conscientious (Rentzsch, Schröder-Abé, & Schütz, 2013). In view of the potential importance of halo effects and other imputed traits, we added an exploratory measure of what other traits people with high or low self-control were assumed to have.

## Method

We investigated how hypothetical individuals with high versus low self-control are perceived by others with respect to 1) how much people would like to be with these individuals in different situations and 2) how much people like these individuals, and 3) how people rate these individuals on a list of traits. We used vignettes in which several aspects were systematically manipulated. As suggested by Simmons, Nelson, and Simonsohn (2012), we report how we determined our sample size, all data exclusions (if any), all manipulations, and all measures used in the study.

### Participants

To achieve the planned sample size, we shared the online study's link via e-mail messages and social networks. We incentivized people with the chance to win a 50 eur Amazon coupon. Participants who studied at the university at which the study was conducted could alternatively receive course credit. For participants to be eligible for data analysis, they had to be at least 18 years old and have completed the full study.

After we excluded participants who did not complete the questionnaire and participants whose answers showed patterns such as simply checking the same number across all items, the remaining sample size was *N* = 285 (71.9% women, mean age = 28.99 years). This far exceeded our planned sample size of approximately 200 participants. Thus, we conducted a sensitivity analysis for the term for which the study design was the least powerful, that is, within-between interactions (e.g., sex-stereotype hypothesis). Using a repeated-measures analysis of variance (ANOVA) with two groups and two measurements, the minimum effect size needed to be *f* = 0.107 (α = β = .05, *N* = 285, correlation among repeated measures = .5, e = 1), which is close to a small (*f* = 0.100) effect. The sensitivity of the analysis given our sample size was thus good. The power analyses were conducted with G*Power (Faul, Erdfelder, Lang, & Buchner, 2007).

### Materials

Participants were presented with a vignette, which was based loosely on past work (Hertel et al., 2007). The vignette contained the person's name, age, sex, place of residence, hobbies, and a description of the person's level of self-control. We varied the vignette's characteristics in a 2 (self-control: high vs. low) × 2 (sex: male vs. female) × 2 (method factor: items used to describe the person) × 2 (order: high-low vs. low-high) design. The first factor was administered within-subjects.

The manipulation of the stimulus person's self-control used items from the German version of the Brief Self-Control scale (Bertrams & Dickhäuser, 2009; Tangney et al., 2004). We adopted this procedure from Shea et al. (2013). We excluded one item due to a low item-total correlation (*r* < .3; see also Bertrams & Dickhäuser, 2009), another because it referred to the respondent's self-perception, and a third because it invoked the respondent's perception of others. We then divided the remaining 10 items into two groups. To do so, we ordered them by their item-total correlations and assigned them to two groups using an ABBA scheme. The A-group items were thus very similar to the B-group items with respect to their mean item-total correlations (*r*_a_ = .428, *r*_b_ = .414). Finally, to indicate whether the vignettes had high or low self-control, we negated the items. For example, “Sometimes I can't stop myself from doing something, even if I know it is wrong” was modified to be “Sarah can usually stop herself from doing something she knows is wrong” (high self-control) or “Sarah usually can't stop herself from doing something even when she knows it is wrong.”

The person was either called Jan (male) or Sarah (female), which are the most common names for German people born in 1992. Their age was 25 in both versions (the study was conducted in early 2018). In order to reduce demand characteristics, we also varied their places of residence, occupation, and hobbies, for example, Jan lived in Duisburg, Germany, was an IT specialist, and liked playing football and guitar, and Sarah lived in Bochum, Germany, was a laboratory assistant, and liked aerobics and playing the keyboard. Thereby, we chose similar options: For example, the place of residence was chosen to have a similar number of inhabitants, the occupation was chosen to have a similar level of income, and the hobbies were chosen to be similar with respect to one of them referring to playing an instrument and the other a team sport.

To assess whether the manipulation of self-control was successful, participants were asked to indicate the presented person's level of self-control after each vignette (manipulation check). As dependent variables, we measured how likeable the people in the vignettes were. We asked the participants how much they would like to be in certain situations with the people described in the vignettes. Situations were either duty situations or socializing situations based on the taxonomy by Rauthmann et al. (2014). Which 5 × 2 situations were presented in which category was determined by a pretest for which we developed 7 × 2 situations. The duty situations featured 1) *being in a study group or team*, 2) *proofreading an application*, 3) *transcribing a presentation*, 4) *asking about a presentation*, 5) *doing the final cleaning of an apartment*, 6) *getting advice for a decision*, and 7) *telling about a personal problem*. The socializing situations were 1) *spending the holidays*, 2) *having a conversation at a party*, 3) *partying*, 4) *going for a walk and having a nice conversation*, 5) *eating away from home*, 6) *spending an evening playing games,* 7) *cooking*. After pretesting, Situations 1 to 5 were selected for the main study, respectively. The socializing situations thus all involved having fun and pleasant, short-term interactions, as opposed to having a long-term relationship in which one might have to depend on the other person.

To assess possible halo effects, participants completed the adjective scales by Jacobs and Scholl (2005), which are based on the interpersonal theory of psychiatry by Sullivan (1953/2013). Information about the adequacy of the reliability and validity for the Interpersonal Adjective List can be found in Jacobs and Scholl (2005, pp. 152-153). There are a total of eight categories (in pairs of opposites) with eight adjectives each. The categories were assured-dominant versus unassured-submissive, gregarious-extraverted versus aloof-introverted, warm-agreeable versus cold-hearted, and unassuming-ingenuous versus arrogant-calculating. Finally, participants completed the German translation of the Brief Self-Control Scale (Bertrams & Dickhäuser, 2009; Tangney et al., 2004). Information about the adequacy of the reliability and validity (e.g., strong correlation with students' grade point average) for the Brief Self-Control Scale can be found in Tangney et al. (2004, p. 287). For all scales, we used equidistant anchors ranging from 1 (*disagree*) to 5 (*agree*) as proposed by Rohrmann (1978).

### Procedure

First, participants provided information about their age, sex, and educational status. Then they were presented with the vignette, the manipulation check, the interpersonal adjectives list, and the situations. They were asked how much they would like to be in each of the situations with the person described in the vignette. The procedure was the same for both vignettes. At the end of the study, participants completed the Brief Self-Control Scale (Tangney et al., 2004), described what they thought the purpose of the study was, and provided further demographic data to check for similarity with the descriptions in the vignette (place of residence, occupation, hobbies, field of study, semester).

## Results

The data were analyzed with IBM SPSS Statistics (Version 25.0). The analysis script and data can be found in Supplementary Materials. A summary of the hypotheses and findings is provided in Table 1.

**Table 1:** Summary of the Hypotheses and Results

Partner in a situation depends	Liking of a person depends	A sex stereotype-self-control fit
*H1: Strongly on the situation*	*H1: On the similarity between respondent and target*	H1: Boosts liking
H2: Weakly on the situation	H2: On complementarity between the respondent and target	
*H3: Positivity: on the partner's self-control*	H3: Compensation: On the total self-control of respondent and target	*Has no effect*
*On the similarity between respondent and partner*	H4: Positivity: on the target's self-control being high	
	*Negativity: On the target's self-control being low*	

*Note*. Hypotheses that are supported by theories are marked with “H” and the respective number. Hypotheses that were supported by the data are written in italics.

### Data Quality Checks

#### Manipulation Check

For the item “self-controlled,” which we had inserted into the interpersonal adjectives list, the high self-control vignette (*M* = 4.45, *SD* = 0.84) received substantially higher ratings than the low self-control vignette (*M* = 1.59, *SD* = 0.79), *t*(284) = 40.16, *p* < .001, *dz* = 2.379. The manipulation was thus quite powerful and successful at creating impressions of the stimulus individuals as high or low in self-control.

We further tested whether the different items we used to describe the high and low self-control vignettes and the order of the two vignettes (high-low vs. low-high self-control) had an effect on the ratings. As ratings, we used the duty situation liking because it was most affected by the other variables and thus seemed to be the most sensitive. There were no order or method effects: Whether participants rated the high self-control vignette or the low self-control vignette first did not have an effect (i.e., no order effects), *F*(1, 283) = 1.47, *p* = .226, $\eta_{p}^{2}$ = .005. The specific items, that is, the items from the Brief Self-Control Scale that were used to describe self-control, did not have an effect either, *F*(1, 283) = 0.02, *p* = .892, $\eta_{p}^{2}$ < .001. There was also no effect of the interaction between the level of self-control in the vignette and the items, *F*(1, 283) = 0.61, *p* = .434, $\eta_{p}^{2}$ = .002.

We checked the internal consistencies of all scales to make sure participants had completed the survey conscientiously. Cronbach's alpha for the Brief Self-Control Scale (13 items, nine reversed ones) was good, α = .842, and very close to the values reported by Tangney et al. (2004, p. 287; α = .83 and .85). Internal consistencies for the duty situations scale were excellent (five items, α = .930 for the Sarah vignette and α = .938 for the Jan vignette). The internal consistency of the Socializing Scale was slightly lower but still good (five items), α = .889 and α = .870, for the two vignettes, respectively. The interpersonal adjective scales all had acceptable or good internal consistencies with the lowest value for unassuming-ingenuous (Sarah vignette), α = .754.

### Hypothesis Tests

#### Situation Hypothesis

**Figure 1:**
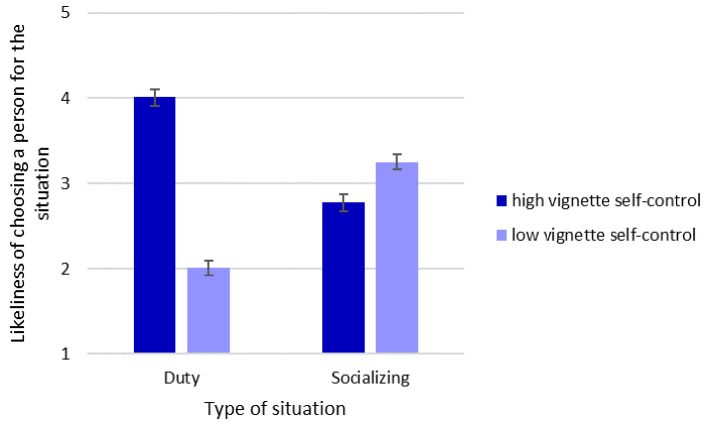
Test of the Situation Hypothesis *Note*. Error bars represent 95% confidence intervals.

To test the situation hypothesis, which, in its strongest formulation claims that people prefer a partner with high self-control in duty situations and a partner with low self-control in socializing situations, we computed a 2 (situation type: duty vs. socializing) × (vignette self-control: high vs. low) repeated-measures ANOVA. There was no main effect of the type of situation, *F*(1, 284) = 0.02, *p* = .897, $\eta_{p}^{2}$ < .001. Overall, partners with high self-control were preferred regardless of the type of situation, *F*(1, 284) = 198.16, *p* < .001, $\eta_{p}^{2}$ = .411. However, there was a significant interaction between type of situation and vignette self-control: Consistent with the strong formulation of the situation hypothesis, people with high self-control were preferred as partners in duty situations but not in socializing situations, *F*(1, 284) = 757.03, *p* < .001, $\eta_{p}^{2}$ = .727. The self-control (dis)advantage was *dz* = 1.706 for duty situations and *dz* = -0.394 for socializing situations. Thus, there was a significant preference for the person with high self-control in the duty situation but also a significant preference for the person with low self-control in the socializing situation (both pairwise comparisons *p* < .001). The results are depicted in Figure 1.

**Figure 2:**
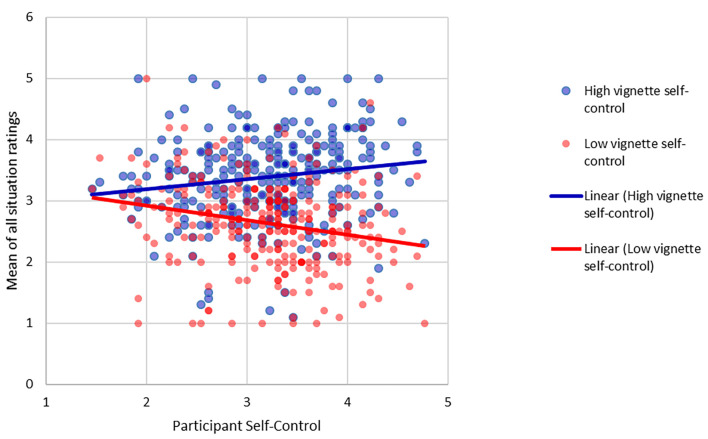
Interaction of Participant Self-Control and Vignette Self-Control on How Likely Participants Were to Choose the Person Described in the Vignette as a Partner in a Variety of Situations *Note*. The solid lines represent regression lines for the respective conditions of vignette self-control (blue: high, red: low).

Besides determining the influence of the type of situation and the vignette self-control, we included participants' self-control in the model for exploratory purposes. The results of the situation hypothesis test did not change. The only additional significant effect was a vignette self-control (duty vs. socializing) × participant self-control interaction effect, *F*(1, 283) = 26.00, *p* < .001, $\eta_{p}^{2}$ = .084, such that participant self-control was positively correlated with the mean of all situation ratings for the high self-control vignettes, *r* = .155, and negatively correlated for the low self-control vignettes, *r* = -.223 (see Figure 2). To sum up, both vignette self-control *and* participant self-control interacted with the type of situation.

#### Liking Hypothesis

**Figure 3:**
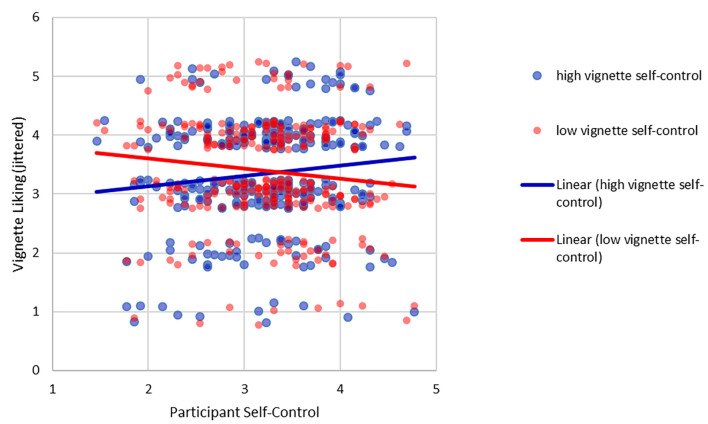
Test of the Liking Hypothesis *Note*. Liking values are jittered for readability (i.e., random noise has been added to the scores to prevent overlap and enhance readability). The solid lines represent regression lines for the respective conditions of vignette self-control (blue: high, red: low).

We tested whether people with high self-control are liked more than people with low self-control and how this interacted with participants' own self-control using an ANOVA with vignette self-control (high vs. low) as a within-subjects factor and participant self-control as a metric factor. There was a main effect of vignette self-control, *F*(1, 283) = 10.43, *p* = .001, $\eta_{p}^{2}$ = .036, such that people with high self-control were liked less than people with low self-control, a finding that was visible only when participants' self-control was controlled for (*M*_liking vignette high self-control_ = 3.34, *SD* = 0.13, *M*_liking vignette low self-control_ = 3.38, *SD* = 0.12, *dz* = 0.155) and not significant when no other variables were controlled for, *t*(284) = 0.49, *p* = .625, *dz* = 0.041. Participants' self-control had no main effect on the ratings, *F*(1, 283) = 0.03, *p* = .863, $\eta_{p}^{2}$ < .001. In line with the similarity hypothesis, participants' self-control moderated the effect of vignette self-control, *F*(1, 283) = 10.20, *p* = .002, $\eta_{p}^{2}$ = .035. The results are depicted in Figure 3. People expressed the highest liking for people most similar to themselves on self-control.

#### Sex-Stereotype Hypothesis

**Figure 4:**
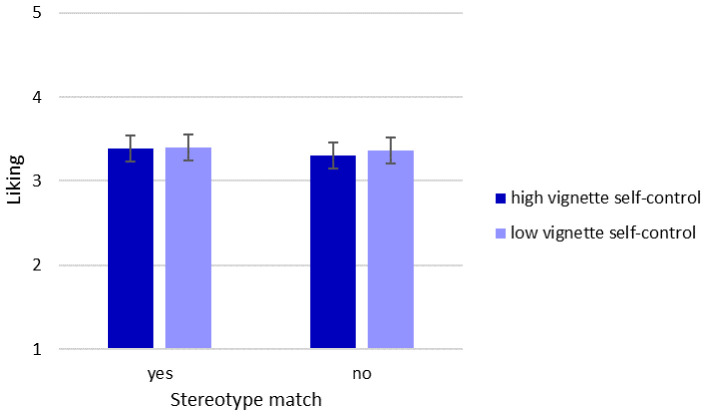
Sex-Stereotype Hypothesis Test *Note*. Error bars represent 95% confidence intervals.

We tested whether a stereotype match (e.g., a man with low self-control or a woman with high self-control) had a positive effect on the extent to which participants liked that person. We computed a 2 (vignette sex: male vs. female) × 2 (stereotype match: yes vs. no) ANOVA. There was no main effect of vignette self-control when we did not control for respondents' self-control, *F*(1, 283) = 0.24, *p* = .622, $\eta_{p}^{2}$ < .001. There was also no main effect of stereotype match, *F*(1, 283) = 0.61, *p* = .435, $\eta_{p}^{2}$ = .002. Finally, there was no interaction between vignette self-control and whether the vignettes matched the stereotypes, *F*(1, 283) = 0.10, *p* = .750, $\eta_{p}^{2}$ < .001. The results did not support the sex-stereotype hypothesis (see also Figure 4).

### Exploratory Analyses

**Figure 5:**
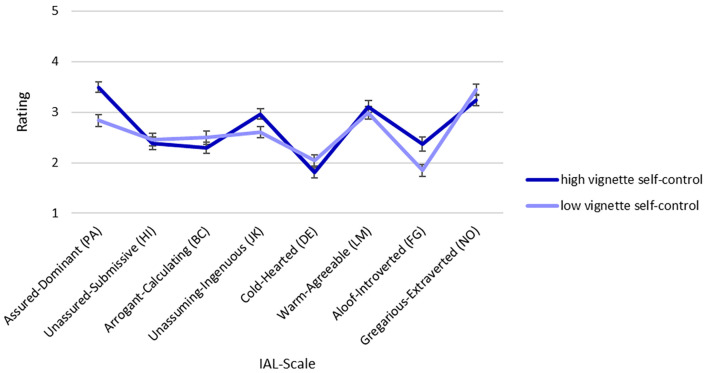
Summary of IAL Ratings for the High and Low Self-Controlled Hypothesis *Note*. Opposite dimensions (e.g., PA vs. HI) have been juxtaposed. Error bars represent 99.375% confidence intervals (Bonferroni corrected).

As we had not proposed specific hypotheses regarding halo effects of self-control on other traits, we investigated this aspect in an exploratory fashion. We ran eight *t* tests (one for each dimension) to determine the effect of vignette self-control. We controlled for an inflation of the alpha level by adjusting the significance level to α = .05/8 = .00625 (Bonferroni correction). Results are depicted in Figure 5 and Table 2. To summarize, almost all facets were sensitive to the manipulations of vignette self-control. People with high self-control were perceived as dominant, unassuming, and introverted, whereas people with low self-control were perceived as arrogant, cold-hearted, and extraverted.^1^1This is surprising given that dominance and introversion are negatively correlated according to the circumplex model. We tested the circumplex model's proposed negative relationships between the respective factors PA-HI, BC-JK, DE-LM, and FG-NO and found the correlations to be much smaller than those reported by Jacobs and Scholl (2005, p. 146). The strongest correlations were between assured-dominant (PA) and unassured-submissive (HI), *r*(283) = -.483, *p* < .001 for the Jan vignettes and *r*(283) = -.514, *p* < .001 for the Sarah vignettes (Jacobs & Scholl, 2005, p. 146 reported *r*[332] = -.84 for self-reports). However, correlations between arrogant-calculating (BC) and unassuming-ingenuous (JK) were very small, *r*(283) = -.099, *p* = .094, for the Jan vignettes and *r*(283) = -.008, *p* = .897 for the Sarah vignettes (Jacobs & Scholl, 2005, p. 146 reported *r*[332] = -.66 for self-reports).


**Table 2:** Differences in Vignettes (High vs. Low Self-Control) on the Dimensions of the Interpersonal Adjective List (IAL)

Dimension	*M*_high vs. low_	*SD*	*dz*	*p*
Assured-Dominant (PA)	0.66	1.06	0.619	< .001
Arrogant-Calculating (BC)	-0.20	0.96	-0.206	.001
Cold-Hearted (DE)	-0.24	0.80	-0.298	< .000
Aloof-Introverted (FG)	0.51	1.11	0.464	< .001
Unassured-Submissive (HI)	-0.07	1.16	-0.058	.325
Unassuming-Ingenuous (JK)	0.36	1.00	0.360	< .001
Warm-Agreeable (LM)	0.12	1.01	0.118	.047
Gregarious-Extraverted (NO)	-0.20	0.99	-0.201	< .001

## Discussion

A broad and high-powered vignette study provided detailed evidence for a wide range of effects of a person's self-control on social perception. Contrary to the view that high self-control is always better than low self-control (positivity hypothesis of self-control), we found that the type of person that is preferred 1) depends on the type of situation one is confronted with, 2) depends on similarity, and 3) does not depend on gender stereotypes. In terms of duty situations (e.g., getting advice for a decision), people with high self-control were the preferred partners, whereas in socializing situations (e.g., going for a walk, attending a party, having a nice conversation), people with low self-control were the preferred partners. Also contradicting the positivity hypothesis of self-control, we found that how much people liked people with low or high self-control depended on their own self-control. More specifically, people with high self-control preferred other people with high self-control, and people with low self-control preferred other people with low self-control, a trend that is consistent with similarity being a major factor for determining attraction in nonromantic contexts.

There was an apparent contradiction of the main effect of self-control: When the task was to choose a partner for socializing and duty situations, participants preferred partners with high self-control overall (high vignette self-control). However, when we asked for how much participants liked the vignettes *and* participants' self-control was controlled for, low vignette self-control received higher liking scores overall. Note that choosing somebody in a situation differs from liking somebody and that the overall mean effect across all 10 situations depended on these situations and could easily be different for other situations. For example, the duty situations might have been more diagnostic than the sociality situations, but at the same time, they might occur less often and thus weigh less.

Although there is evidence for stereotypical male behavior being less self-controlled than female behavior, and there are theoretical grounds for predicting that a stereotype match would be positively associated with how much a person likes another person, we found no such effect: Whether men or women were presented as high or low in self-control had no differential effect on liking.

We found that the similarity effect and the situation effect occurred simultaneously and independently and dominated the main effect of vignette self-control (positivity hypothesis) by far. That is, the choice of a partner depends on the situation that the partner is needed for (situation hypothesis), the resemblance between the partner and the person who is choosing the partner (complementarity hypothesis), but not or only to a very small degree on the self-control of the partner being high (positivity hypothesis). Future research should consider these aspects of social perception in relation to self-control. We did find an advantage of people with high self-control over people with low self-control when people look for partners in duty situations. Despite this corroboration of the positivity hypothesis, other factors such as similarity and the situation seem more important. This is surprising given that the definition of self-control includes aspects of social desirability. In other words, whether one likes somebody who is socially desirable (i.e., has high self-control) also depends on the perceiver's self-control. And whether one wants to work with somebody who is socially desirable depends on the situation. Ironically, in what we termed *socializing situations* (e.g., going on vacation together or partying), vignette characters with low self-control (i.e., a socially undesirable trait) were preferred over vignette characters with high self-control.

Finally, and more in line with the positivity hypothesis of self-control, an exploratory approach revealed that people with high self-control are associated with a wide range of other socially desirable traits, such as being assured, unassuming, and agreeable. Some of the results are seemingly contradictory: Although some factors theoretically correlate negatively (e.g., assured-dominant and gregarious-extraverted), differences between high and low self-controlled vignettes are not in the same direction with these factors. This could be due to patterns that are different in that population, possibly due to trans situational variability, than in a standard population. That is, people very high and low in self-control or the perceptions about these people could show other patterns of correlations between the dimensions that differ from those in a standard population.

### Limitations

In our outline of the social perception of people with high self-control, we considered different kinds of situations, individual differences in the participants, sex stereotypes, and a wide range of traits. However, several factors merit further investigation.

First, we used vignettes only. Our setting was thus highly artificial, and the large effect sizes were probably due to the extreme manipulations. If actual people with less extreme differences were used, we would expect smaller effects.

Second, our study may have been affected by demand characteristics. It was evident that we were interested in perceptions that were based on the vignettes and on stereotypes. Even though people are not defenseless against demand characteristics (Hofmann, Gschwendner, Friese, Wiers, & Schmitt, 2008) judgments in everyday life may differ from findings in an experiment. The ratings of the vignettes might be subject to demand characteristics whereas spontaneous responses might not. Although tasks such as the Implicit Association Test (Greenwald, McGhee, & Schwartz, 1998) have been criticized heavily (e.g., Schimmack, 2019), they allow for an assessment of such spontaneous reports (Hofmann, Gawronski, Gschwendner, Le, & Schmitt, 2005) and can thus be helpful to avoid the problem of demand characteristics.

Third, our sample and vignettes targeted 20- to 30-year-old German people. Academic and social goals may vary across the lifespan and cultures, moderate the importance of duty and socializing situations, and thus affect their weight in choosing interaction partners and evaluating others. Even what is socially desirable might depend on these factors. For example, an interesting approach could be the longitudinal perspective on romantic relationships with respect to self-control. At the beginning of a romantic relationship, partners might exercise much more self-control in order to convince their partner of sustaining the rather fragile relationship than after a few months or years.

Although we identified situation type, similarity, and self-control as three parallel effects, with the last one being the weakest in our study, we cannot generalize their significance to contexts outside the laboratory. Generalization to other cultures where different traits are considered socially desirable may not be possible either. Replications, especially in non-Western cultures, are needed. Due to the artificial setting and different manipulations and measures (i.e., manipulation of vignette self-control but no manipulation of respondent self-control), the effect sizes might be skewed.

### Conclusion

Although self-control is usually understood as a socially desirable trait that is associated with functional behavior, we provided evidence against the simple assumption that more self-control is always better. Although there was a slight overall preference for people with high self-control, these preferences changed significantly (and even occasionally reversed) on the basis of similarity and type of situation. Our findings may be reassuring for people who have low self-control because they show that such people will sometimes be liked and chosen ahead of others. Sometimes, at least, it is advantageous to lack self-discipline.

## Data Availability

Data for this article is freely available (see Röseler, Ebert, Schütz, & Baumeister, 2021).

## Notes

For this article the following Supplementary Materials are available via the PsychArchives repository (for access see Index of Supplementary Materials below):

- Data analysis.
- Materials from the experiment.
